# Practical and Ethical Concerns in Implementing Enhanced Surveillance Methods to Improve Continuity of HIV Care: Qualitative Expert Stakeholder Study

**DOI:** 10.2196/19891

**Published:** 2020-09-04

**Authors:** Mara Buchbinder, Colleen Blue, Stuart Rennie, Eric Juengst, Lauren Brinkley-Rubinstein, David L Rosen

**Affiliations:** 1 Department of Social Medicine Center for Bioethics University of North Carolina at Chapel Hill Chapel Hill, NC United States; 2 Institute for Global Health and Infectious Diseases University of North Carolina at Chapel Hill Chapel Hill, NC United States; 3 Department of Social Medicine Center for Health Equity Research University of North Carolina at Chapel Hill Chapel Hill, NC United States; 4 Division of Infectious Diseases Department of Medicine University of North Carolina at Chapel Hill Chapel Hill, NC United States

**Keywords:** HIV surveillance, retention in HIV care, qualitative research, public health ethics

## Abstract

**Background:**

Retention in HIV care is critical to maintaining viral suppression and preventing further transmission, yet less than 50% of people living with HIV in the United States are engaged in care. All US states have a funding mandate to implement Data-to-Care (D2C) programs, which use surveillance data (eg, laboratory, Medicaid billing) to identify out-of-care HIV-positive persons and relink them to treatment.

**Objective:**

The purpose of this qualitative study was to identify and describe practical and ethical considerations that arise in planning for and implementing D2C.

**Methods:**

Via purposive sampling, we recruited 43 expert stakeholders—including ethicists, privacy experts, researchers, public health personnel, HIV medical providers, legal experts, and community advocates—to participate in audio-recorded semistructured interviews to share their perspectives on D2C. Interview transcripts were analyzed across a priori and inductively derived thematic categories.

**Results:**

Stakeholders reported practical and ethical concerns in seven key domains: permission and consent, government assistance versus overreach, privacy and confidentiality, stigma, HIV exceptionalism, criminalization, and data integrity and sharing.

**Conclusions:**

Participants expressed a great deal of support for D2C, yet also stressed the role of public trust and transparency in addressing the practical and ethical concerns they identified.

## Introduction

Retention in HIV care is critical to maintaining viral suppression and preventing further transmission, yet less than 50% of people living with HIV in the United States are engaged in care, and only 56% are virally suppressed [1]. Common barriers to remaining in care include: feeling depressed or stigmatized; substance use; low literacy; day-to-day responsibilities, including work or school; inadequate housing, insurance, and related financial problems; lack of reliable transportation, particularly for rural populations; and institutional variability in attempts to contact and locate patients who miss appointments [2-6]. Low care engagement results in excess morbidity and mortality among people living with HIV and fuels HIV transmission [7]. Accordingly, identifying out-of-care people living with HIV and linking them to sustainable care is essential to addressing the HIV epidemic.

As interconnecting sources of electronic data expand, state and local health departments are increasingly pursuing novel strategies, including health information technologies, to re-engage out-of-care people living with HIV in care [8,9]. All states in the US have a funding mandate to implement Data-to-Care (D2C) programs, which use surveillance data such as HIV viral load test results, Medicaid claims records, or electronic health records from private or state-run systems to identify out-of-care HIV-positive persons and re-link them to treatment. Because HIV viral load test results are mandatorily reported to public departments of health (DOH), they can be used to assess retention in care.

In the DOH model of D2C, which we focus on here, the first step is for a state or local DOH to use its surveillance data to generate a list of people living with HIV identified as being out of care. Typically, this is defined as someone who has not had a viral load laboratory test reported in the previous 12 months. Because misclassification of care status can occur while using reported viral load test results due to delays and incomplete reporting [10], additional data sources, such as state Medicaid records, electronic health records, or mortality records, may be checked to verify whether a person is not in care. Public health personnel can then contact the patient’s last known HIV provider. If the patient is confirmed to be out of care, the health care provider may try to contact the patient, or a specially trained public health outreach worker employed by the DOH may reach out to the patient, either by telephone or in-person. The outreach worker or health care provider will then assess whether the patient is indeed out of care, and if so, why. The goal of this contact is to help patients overcome any barriers so that they can resume care [11].

Preliminary research suggests that D2C activities are effective at re-engaging out-of-care individuals in care [12-14], yet few studies have examined the practical and ethical issues raised by such novel applications of health information technologies [15-17]. For example, physicians have expressed concerns about DOH personnel intruding on patient privacy and the physician-patient relationship [18]. The purpose of this study was to identify and describe practical and ethical considerations that arise in planning for and implementing D2C.

## Methods

### Overview

This article reports findings from a larger qualitative study of expert stakeholders’ perspectives on the potential to use criminal justice system data to enhance surveillance and D2C to understand and improve continuity of care among people living with HIV/AIDS in North Carolina who have spent time in county jails. For this sub-study, we focus on stakeholders’ reported views on the current use of D2C in the general population. The Institutional Review Board of the University of North Carolina at Chapel Hill approved the study. Below we describe our methods for recruitment, data collection, and analysis. A full description of the parent study, data collection, and analysis are provided elsewhere [19].

### Data Collection

Expert stakeholders were recruited via a purposive sampling strategy in which we aimed to recruit three to five participants in several categories of professional expertise (public health, ethics and privacy, legal experts and criminal justice personnel, and community advocates). Potential participants were identified using a combination of methods, including the research team’s professional network, literature review and online searches, and snowball sampling. Because the larger study was focused on applications of enhanced surveillance methods and D2C to North Carolina jails, we oversampled expert stakeholders located in North Carolina. Prospective participants were invited over email to participate in the study.

Semistructured interview guides included questions about the participant’s professional background and perspectives on HIV surveillance and D2C in the general population, the potential use of HIV surveillance and D2C in North Carolina jails, privacy, community engagement, data governance, and research practices. In some cases, guides were further tailored to stakeholder categories to collect specific information. For example, DOH personnel were asked additional questions about D2C operations. This article focuses on participants’ responses to questions about HIV surveillance and D2C in the general population. Three members of the research team with training in qualitative interviewing conducted all interviews after obtaining informed consent. Except for one participant, interviews were audio-recorded and conducted either in person (n=28) or via videoconference (n=12) or telephone (n=3). Interviews were conducted between April 2018 and August 2019 and lasted between 40 and 107 minutes.

### Data Analysis

We used Dedoose software to analyze interview transcripts across twenty-two thematic codes. After coding was completed using a set of procedures reported elsewhere [19], we identified salient themes for further analysis and further examined coding reports from each coding category to identify patterns across the larger dataset. For this article, we focused on stakeholders’ responses in seven thematic domains relevant to practical and ethical concerns in implementing D2C: permission and consent, government assistance vs overreach, privacy and confidentiality, HIV stigma, HIV exceptionalism, HIV criminalization, and data integrity and sharing. For this substudy, we excluded responses from four jail administrators, whose expertise was not relevant to this analysis.

## Results

Forty-three expert stakeholders—including ethicists, privacy experts, researchers, public health personnel, HIV medical providers, legal experts, and community advocates—participated in this sub-study (see Table 1). The majority of participants came from North Carolina (26/43); the remainder lived in other states (n=15) or outside the United States (n=2). Participants universally acknowledged the public health needs that DOH-based D2C programs aim to address, and most expressed support for the public health goals such programs fulfill. As one participant put it, “I feel if you have a public health imperative and you can do things about that, and you can treat and basically save people’s lives, that you have a responsibility to try to do that.” In discussing the practical and ethical considerations of implementing such programs, however, participants qualified their support with a range of significant concerns, which clustered into the seven themes identified above. Below, we describe findings from each theme in more detail. We offer illustrative quotations from stakeholders in Table 2.

**Table 1:** Stakeholder type (N=43).

Stakeholder categories		Count, n
**Ethics and privacy**		
	Ethicists	4
	Privacy experts	5
**Public health**		
	Public health researchers	8
	Federal, state, and local public health personnel	8
	HIV linkage staff	4
	Community HIV providers	4
Legal experts		3
**Community advocates**		
	Criminal justice advocates	3
	HIV community advocates	4

**Table 2:** Illustrative quotations.

Concept	Participant comments
Permission and consent	*Permission should be obtained*: “Absolutely they should have permission…People might not even know that they’re in a surveillance system. And given the potentially adverse consequences of the use of that data, there might be cause for concern there for those folks.” (1010, privacy expert) *Obtaining permission would impede public health objectives*: “Probably hard to have a program like this that’s opt in. When you do that, either no one does or the only people who do are people getting treatment already.” (1035, privacy expert)
Government assistance vs overreach	*D2C*^a^*is justified assistance*: “So, for HIV we intercede, we stick our noses into people's medical records extensively with the practical goal of making sure people receive treatment, which is not ethically a bad goal, but it can be highly intrusive, although we try to do it sensitively, and is not everybody's personal goal.” (1002, public health employee) *D2C is government overreach*: “There are a lot of things that people do or don’t do that affect health or wellbeing or whatever and the state could intervene with them to say do better…Kind of a nanny state.” (1035, privacy expert)
Privacy and confidentiality	*Health worker showing up could breach confidentiality*: “[A] state health official showing up could alert family members, could alert folks in the neighborhood, could alert others in the household. Hey, there’s something. We’re not sure what, but there’s something going on.” (1011, researcher) *People may not want to be contacted*: “[There’s] reasons people are not in this care continuum. And they may not want to be found. They may think finding them will bring other kinds of surveillance that they’re not interested in having.” (1013, ethicist)
HIV stigma	*D2C could exacerbate stigma*: “The way that [the D2C] system works, I don’t see that as helpful, because you’ve got these strange people knocking on your door looking for you, and you don’t really understand who these strange people are. And because these people are appropriately afraid of the system they always think somebody’s coming after them to incarcerate them, to take them to court. So that deepens the stigma.” (1036, HIV Provider)
HIV exceptionalism	*Not problematic that D2C is focused on HIV*: “HIV is exceptional because HIV is different. And it’s exceptional in lots of ways. So, our response to it has to be exceptional in some ways.” (1030, ethicist) *D2C focus on HIV is stigmatizing*: “It almost seems stigmatizing in the way that [HIV] is so singled out and so hyper focused on. Not that it doesn’t deserve that amount of focus and resources, but that it’s to the exclusion of other things…A job, etc.” (1033, criminal justice advocate)
HIV criminalization	*D2C could lead to punitive measures for PLWH*: “The community doesn’t see it that way. They see [D2C] as a way that will create opportunities for criminalization, that it can be used against people. (1021, HIV advocate)
Data integrity and sharing	*D2C increases risk of data reaching “the wrong hands”*: “I would say that there are probably potentially more risks because, as more data changes hands, there’s always the possibility that it could end up in the wrong place or in the wrong hands.” (1031, public health personnel) *Data could be misused*: “I think that the fears that the individuals have that the data will be used in some other way that the—I don’t want to say criminal, but certainly the people in government might start misusing those data in ways that were not intended from big data work for that. And then the current environment, governmental environment in the country I think that that fear is incredibly reasonable.” (1047, HIV advocate)

^a^D2C: data-to-care.

### Permission and Consent

Stakeholders were largely divided by stakeholder type on whether permission and consent for D2C should be obtained. Those in favor of obtaining consent for future contact associated with D2C at the time of diagnosis—including most privacy and legal experts, community advocates, and some ethicists—argued that doing so would demonstrate respect and dignity, improve the government’s credibility, and that the risks of public harm created by potential refusals were too low to justify overriding consent on public health grounds. However, even those who thought consent should be obtained acknowledged the practical challenges of doing so, and that permitting people to opt out would potentially impede the efficacy of D2C. Others—including most public health personnel, researchers, and some ethicists—argued that forgoing consent was justified because D2C is a core component of public health surveillance, which does not require consent. They argued that obtaining consent would limit the state DOH’s ability to intervene and that the state should act on this information to return out-of-care patients to care rather than do nothing. One public health employee noted that if surveillance is to proceed without informed consent, treatment must be non-coercive. Several others suggested that in lieu of consent, the DOH should inform people that D2C is occurring, ideally through providers’ offices. One researcher suggested this is best framed as a way to support people living with HIV, rather than a response to “falling out of care.”

### Government Assistance Versus Overreach

Five public health personnel emphasized that the state DOH has a responsibility to the public to implement D2C, even at the expense of some individual privacy. They argued that the agency’s public health mission and legal authority provide adequate justification for the level of state intrusion required for D2C, as long as the right to refuse care is ultimately preserved. On the other hand, 11 stakeholders, particularly ethicists, researchers, privacy experts, and some public health personnel, thought that people would object to the state tracking them or contacting them about their healthcare through surveillance and D2C, and some thought that this might constitute an unwelcome form of government intrusion. Six of them explicitly suggested that such activities reflected the work of “Big Brother” or a “nanny state.” Overall, participants expressed concerns about the potential for government overreach more frequently than they defended the necessity of this type of assistance. Nevertheless, ten stakeholders still thought the benefits of D2C outweighed the risks of government overreach, and several had suggestions for how to mitigate these concerns through implementation procedures.

### Privacy and Confidentiality

Stakeholders uniformly acknowledged that a health worker showing up at someone’s home as a result of D2C activities could constitute an unwanted invasion of privacy by alerting family members or neighbors to a potential problem. Four noted that these types of privacy concerns might be more pronounced in areas with heightened HIV stigma (see below), particularly in rural areas, and that these violations could have serious ramifications for trust in government. Eight stakeholders saw such intrusions into private space as a more significant violation because people living with HIV may not want to be contacted for linkage to care and have a legal and ethical right to refuse care. One public health employee suggested that this type of privacy violation is especially significant in the D2C context because informed consent is not obtained, and HIV surveillance data is being used differently from its initial authorized purpose, which was purely for tracking rather than recontacts and linkage to care.

The potential for inadvertent disclosure was the biggest concern associated with D2C. Stakeholders displayed different levels of trust that private health information collected as a result of D2C will remain confidential. DOH personnel noted that community health workers are very well-trained, suggesting a low probability for disclosure, while privacy experts averred that the risk of a breach increases with more people accessing confidential information, regardless of the context. Several HIV providers reported that their patients had had negative experiences with disease intervention specialists (DOH employees who contact people newly diagnosed with HIV to collect information about potential contacts and risk factors and to help connect them to care) at the time of diagnosis. These experiences suggested to these HIV providers the potential for a breach of confidentiality by DOH outreach workers engaged in D2C. One researcher viewed sharing confidential information with health workers as a breach in itself. Four stakeholders suggested that the risk of a breach may be greater in rural communities where there may be a greater risk of overlap in the social networks of health workers and the communities they serve.

### HIV Stigma

Many stakeholders suggested that the public response to D2C depended in part on HIV stigma. While some stakeholders believed that HIV stigma has decreased over time, others—particularly HIV providers—still see evidence of substantial stigma (eg, patients traveling far away from their home communities to access HIV care or choosing to forego care). Fourteen participants mentioned that D2C could potentially heighten HIV stigma through unwanted attention from state health workers, privacy violations, and inadvertent disclosure, yet varied in terms of how likely they viewed this scenario. Concerns about this possibility were embedded in broader concerns related to the marginalization of vulnerable groups (eg, African Americans, men who have sex with men, and transgender people) and HIV exceptionalism (discussed further below). Three stakeholders cautioned that D2C could be implemented in a way that alienates people from systems of care, produces panic, or overlooks the circumstances of people’s lives in ways that reinforce stigma.

### HIV Exceptionalism

HIV exceptionalism is the view that, for a variety of reasons, HIV is or should be treated differently than other communicable diseases or conditions that may result in death if untreated. D2C may be an example of HIV exceptionalism because it is used widely for HIV, but much less commonly for other conditions. Stakeholders were overall split regarding whether it is problematic for D2C to focus on HIV, with many people remaining uncertain. Six participants raised the possibility that HIV exceptionalism heightens stigma, and four suggested that if there were similar surveillance-based interventions for other conditions, it might reduce some of the stigmas around HIV because people would not feel singled out for their HIV status. Ten participants indicated that D2C should be used for other conditions, especially infectious or sexually transmitted diseases.

### HIV Criminalization

When asked about possible risks or harms of HIV surveillance and D2C, 12 stakeholders mentioned the possibility that D2C could lead to punitive measures for people living with HIV. Some state laws require people living with HIV to disclose their HIV status to partners if they are not virally suppressed. One ethicist stated that HIV surveillance is necessarily problematic in a context in which HIV is criminalized. At the same time, a community HIV advocate noted that the potential for criminalization could be used to try to persuade people living with HIV who have fallen out of care to re-establish care.

### Data Integrity and Sharing

Data integrity is a basic tenet of public health surveillance because there are always increased security risks when using and sharing data. Many participants expressed concerns that D2C programs could inadvertently result in sensitive personal information reaching the “wrong hands,” particularly in rural areas. Possible risks of someone outside of DOH personnel illegally obtaining data include data breaches and malware attacks. Four stakeholders, including a privacy expert, community advocate, and two legal experts, raised concerns about the possible harms that might occur if D2C personnel obtained erroneous data. For example, incorrect data could lead state health workers to contact the wrong person for re-engagement in care. Nevertheless, public health personnel reported that wrongful identification, although possible, was rare due to rigorous data cleaning and matching before field contacts are attempted.

Four stakeholders mentioned concerns about possible misuse of the data by the government—for example, suggesting that the information might be shared with legislators to enhance criminalization laws. Several stakeholders noted that many people do not understand or trust data protections and that the government sponsorship of D2C increases mistrust, especially among African American communities. Stakeholder recommendations included: creating oversight for how data is collected, used, and shared, including necessary safeguards to protect against breach or misuse, checks and balances to ensure the data is accurate, and strong security measures.

## Discussion

Expert stakeholders expressed a range of ethical and practical concerns related to the use of D2C to improve the continuity of HIV care. Most stakeholders acknowledged that using big data methods to re-engage patients in care is a logical extension of public health surveillance that is justified by the mission of state and local health departments to reduce HIV transmission and promote public health. At the same time, D2C also represents a new application of existing surveillance data that may raise the suspicions of some community members [20,21]. The tension between government assistance and government overreach encapsulates the promise and pitfalls of using D2C and other big data technologies in public health interventions.

Responses from expert stakeholders emphasized that context matters greatly to the ethics of D2C. Many stakeholders suggested that privacy and stigma concerns are more pronounced in areas of the rural south where many study participants are located and among vulnerable groups such as racial, ethnic, and gender and sexual minorities. Our findings lend additional support to previous studies suggesting that stakeholder engagement in program implementation is critical for ensuring that D2C programs and other public health surveillance programs are designed in contextually sensitive ways [22,23], particularly given the high degree of support for the notion that D2C could heighten stigma. The public response to digital surveillance has demonstrated this point during the COVID-19 pandemic, which may reinforce the distrust of public health authorities [24].

At the same time, a few stakeholders expressed caution about community engagement. Two participants noted that some people might feel exploited if the motivations for engagement are not genuine, and one suggested that community engagement may inadvertently lead to the spread of misinformation. These findings suggest that care must likewise be taken concerning data protection and data stewardship, both to safeguard against potential breaches and to ensure the trust of the community. Such efforts can mitigate potential mistrust of government motives regarding D2C and the necessary privacy violations entailed. While conducting HIV surveillance without individual informed consent has been ethically justified [15,16,25], the strength of concerns expressed by several stakeholder groups (eg, community advocates, privacy and legal experts) about the lack of informed consent highlight the importance of making communities aware of these public health activities and the reasons for forgoing consent. Such public transparency is a critical component of stakeholder engagement as D2C continues to evolve.

The strengths of this study include its qualitative design, which is well equipped for capturing rich descriptive information regarding practical and ethical challenges in implementing new surveillance methods. Interviews captured nuanced expert perspectives from a wide range of disciplinary backgrounds. The primary limitation is that the purposive sample may not reflect the breadth of views about D2C from all relevant stakeholders. Because the majority of participants came from North Carolina, and public health resources vary widely by state, studies based in other locations may raise different issues. Our interviews focused on the DOH model of D2C. Thus, findings may not be generalizable to other models, such as the use of patient registries generated by specific health care systems.

### Conclusions

This qualitative, descriptive study contributes valuable information that will be useful for understanding future applications of D2C and related surveillance methods. Participants expressed a great deal of support for D2C, yet also stressed the role of public trust and transparency in addressing the practical and ethical concerns they identified. The next steps for the ongoing expansion of D2C programs are pre-implementation community engagement efforts to foster public trust and transparency.

## Abbreviations

D2C: data-to-care
DOH: department of health
